# Retraction: Uterine Inversion Secondary to a Large Prolapsed Leiomyoma: Diagnostic and Management Challenges

**DOI:** 10.7759/cureus.r29

**Published:** 2021-04-14

**Authors:** Anastasia Martin, Anastasios Tranoulis, Ahmad Sayasneh

**Affiliations:** 1 Obstetrics and Gynaecology, King's College London - School of Medicine, London, GBR; 2 Gynaecological Oncology, Guy's and St Thomas' NHS Foundation Trust, London, GBR; 3 Gynaecological Oncology, Guy's and St Thomas' Nhs Foundation Trust, London, GBR

This article has been retracted and removed due to a formal request from the authors made at the behest of the patient featured in the case. No evidence of any author misconduct has been found, however, the authors wish to honor the patient's request. The retraction request from the authors has been included below:

As the most senior academic author on the paper, and in agreement with my other co-authors, we formally wish to request the retraction of this published article. We have reached this sad decision after long discussions among ourselves as authors and after consulting the academic board in the Gynaecological Oncology Department at Guy’s and St Thomas’ NHS Foundation Trust.

Our patient gave consent for her case report to be published while she was in the hospital after a successful surgery and excellent recovery. The initial consent form signed was the surgical consent for the procedure itself which allowed us to take photos for teaching and the second informed written consent form was a standard form for publication in a journal. At that time she was very happy for the case to be published. A year and a half later she wrote to me expressing that although she signed the consent form she did not read the article before publication and she requested a copy of the publication. Later she wrote expressing her wish for the publication to be withdrawn based on that she did not consent for this particular paper. Different reasons were given in subsequent conversations but she remained adamant that the paper be removed.

Although some might argue that we can challenge these allegations, our ethical commitment to good clinical practice and putting patients first has led us to formally request the retraction of this article.

